# The Dark Tetrad and academic dishonesty: a systematic review and narrative synthesis of personality predictors of cheating, plagiarism, and deception in education

**DOI:** 10.1186/s40359-026-04894-8

**Published:** 2026-05-29

**Authors:** Ben Jones, Lee Jones

**Affiliations:** 1 School of Health, Psychological, and Medical Sciences, University of Southern Queensland (UniSQ), Ipswich, Australia; 2 Adaptive Biotoxicology Lab, School of the Environment, University of Queensland (UQ), Brisbane, Australia

**Keywords:** Dark Tetrad, Machiavellianism, Narcissism, Psychopathy, Sadism, Academic Dishonesty, Cheating, Plagiarism

## Abstract

**Background:**

The subclinical personality traits of Machiavellianism, narcissism, psychopathy, and everyday sadism (i.e., the Dark Triad/Tetrad) have been linked to a range of antisocial and deceptive behaviours. With increasing concern about integrity in higher education, it is important to understand how these traits relate to academic misconduct with AI-assisted misconduct becoming more commonplace.

**Methods:**

This systematic review and narrative synthesis examined evidence from 23 studies that investigated associations between dark traits and dishonest academic practices, including plagiarism, exam cheating, contract cheating, academic dishonesty, and AI-assisted cheating.

**Results:**

Psychopathy showed the most consistent pattern of associations across misconduct behaviours. Machiavellianism was associated with some forms of dishonesty, such as plagiarism and cheating, but was not consistently associated with contract cheating. Narcissism showed weaker and more context-dependent associations. Everyday sadism was examined in comparatively few studies. Preliminary evidence linked sadism to some forms of academic dishonesty, including lying in academic contexts and AI assisted misconduct, but the small evidence base means that conclusions about its contribution to the Dark Tetrad framework remain tentative.

**Conclusions:**

Further research is needed before stronger conclusions can be drawn about sadism’s role in academic misconduct. Future directions and implications, including suggestions for replication and expansion, are discussed.

**Supplementary Information:**

The online version contains supplementary material available at 10.1186/s40359-026-04894-8.

## Background

Academic misconduct (cheating, plagiarism etc. in an academic context) has become an increasingly pressing concern within higher education. The widespread availability and rapid adoption of generative AI tools such as ChatGPT have made outsourcing of academic work to AI more accessible and straightforward than ever before with minimal effort [1]. Historically associated with general cheating behaviour are the darker personality traits, commonly linked with callous, manipulative, and self-serving tendencies. The primary aim of this paper is to conduct a thorough systematic review and narrative synthesis examining existing evidence on the relationship between dark personality traits and academic misconduct, thus providing a clearer understanding of the personality factors that may influence dishonest academic behaviours.

### The Dark Tetrad

The original Dark Triad^1^ is composed of three socially undesirable and overlapping traits, these being narcissism, psychopathy, and Machiavellianism [2]. The most recent addition to the Dark Triad is sadism (everyday sadism), forming the Tetrad [3]. These traits exist in line with other models of personality such as the HEXACO model [4] but manifest differently, typically in antisocial behaviour. These traits are considered non diagnostic, subclinical, and manifest as traits on a spectrum in the general population [5]. This distinguishes them from the clinical population and leaves them as functioning members of society [6] despite eliciting socially undesirable behaviour.

Narcissism is a complex and multifaceted construct and is often associated with grandiosity, self-importance, and entitlement [7]. Researchers typically distinguish narcissism into grandiose and vulnerable dimensions [8]. Grandiose narcissism is characterised by overt expressions of superiority, entitlement, dominance, and a strong need for admiration [9, 10]. Individuals high in grandiose narcissism often display confidence, aggression, and low agreeableness, and may engage in manipulative or exploitative behaviours to maintain their inflated self-image [11]. In contrast, vulnerable narcissism involves hypersensitivity to criticism, insecurity, social withdrawal, and feelings of inadequacy behind a façade of self-importance [11]. These individuals often appear defensive, anxious, and resentful, and more prone to internalising problems such as depression or anxiety [12]. These characteristics often lead to antisocial behaviours such as aggressive behaviour when receiving criticism and cognitive overconfidence [13, 14].

Subclinical psychopathy refers to psychopathic traits found in the general population, generally absent of the severe antisocial behaviours and legal consequences associated with clinical psychopathy [15]. It is commonly divided into two distinct but related components: primary psychopathy, characterised by callousness, lack of empathy, superficial charm, and emotional detachment; and secondary psychopathy, marked by impulsivity, emotional dysregulation, and heightened reactivity to stress [16]. These components manifest differently in distinct behavioural patterns, primary psychopaths are more likely to engage in calculated risk-taking, manipulative interpersonal behaviour, and are generally resistant to anxiety or guilt [17]. Conversely, secondary psychopaths display greater impulsivity, reactive aggression, and social deviance [18]. Psychopathy is a core component of the Dark Triad but is often considered the more malevolent of the three due to its callousness and antisocial behaviour [2].

Machiavellianism is associated with manipulative behaviours in the form of personal gain, immoral and pragmatic beliefs, and lack of emotionality [19]. Individuals high in Machiavellianism tend to use deception, charm, and calculated tactics to exploit social situations, frequently prioritising long-term goals over immediate gratification [20]. Unlike psychopathy, which is associated with impulsivity and emotional dysregulation, Machiavellianism is marked by cold, deliberate planning and emotional detachment and more likely to engage in lying, flattery, strategic manipulation, and unethical decision making [9, 21]. There is high overlap between psychopathy and Machiavellianism, especially for manipulativeness, but psychopathy distinguishes itself due to emotional deficits and impulsivity [9] and this has linked it to several behaviours such as bullying, immorality, and cheating [22–24]. Continued research has endeavoured to reduce the conceptual overlap of Machiavellianism with psychopathy, emphasising its unique emphasis on calculated manipulation over impulsivity or aggression [20].

Everyday sadism refers to the tendency to derive enjoyment from the physical or psychological suffering of others and has been conceptualised across behavioural domains including physical aggression, verbal aggression, and the consumption of violent media [25]. Unlike clinical sadism, which is often associated with sexual gratification or criminal behaviour, everyday sadism is subtler and may manifest in socially acceptable or normalised forms such as enjoying violent media, humiliating others online, or taking pleasure in others’ discomfort [26, 27]. Sadism was introduced as the fourth component of the Dark Tetrad to account for a distinct form of malevolent behaviour that was not fully captured by narcissism, Machiavellianism, or psychopathy [28], with sadism displaying unique variance in antisocial behaviour, especially where cruelty is gratuitous or motivated by pleasure rather than personal gain or reactive aggression [29]. Sadists are more likely to seek out opportunities to harm others, even when doing so incurs no instrumental benefit, a pattern not typically observed in psychopathy or Machiavellianism [30]. As a result, it has been linked to behaviours such as trolling [31], counterproductive work behaviour [32], and vindictive lying [33]. Although the present review focuses on studies measuring multiple dark traits to allow comparison, it is worth noting that a substantial body of research has examined each trait individually as noted above for the trait subtypes (e.g., grandiose narcissism, secondary psychopathy etc.). For example, narcissism has been linked to self-enhancement and entitlement motives, psychopathy to impulsive and callous antisociality, Machiavellianism to strategic cheating and manipulation, and everyday sadism to the enjoyment of cruelty and norm violation [27, 34–36]. These single trait literatures provide an important theoretical backdrop for understanding how the traits may translate into academic misconduct.

Collectively, the Dark Tetrad traits can be understood as occupying distinct “immoral profiles” [6, 27, 37]. Machiavellianism is primarily instrumental and strategic, emerging in calculated, long-term rule breaking aimed at personal gain. Narcissism tends to reflect ego-protective and self-enhancing motives, with unethical behaviour occurring when self-esteem, status, or entitlement are threatened. Psychopathy reflects impulsive and callous antisociality, often involving risk-taking and a diminished sensitivity to punishment or moral constraints. Sadism is unique in that norm violation may be intrinsically rewarding rather than instrumental. These profiles provide a theoretical basis for hypothesising differential associations between traits and particular forms of academic misconduct. In addition to their distinct immoral profiles, prior research suggests that the Dark Tetrad traits share partially overlapping behavioural expressions. For example, counterproductive work behaviours and workplace deviance are reliably associated with all four traits, indicating a common propensity towards norm violation and low agreeableness [38]. However, the strategies underlying norm violation differs across traits. Psychopathy has been conceptualised in evolutionary accounts as a social cheating, fast life history strategy involving exploitation of cooperative systems, low empathy, and a reduced concern for punishment [39–41]. Machiavellianism, by contrast, is better characterised by deliberate, long term exploitation of cooperative systems, strategic manipulation, and instrumental exploitation, particularly when such behaviours can be planned and reputational risk managed [19, 42], whereas psychopathy reflects a more impulsive, risk taking approach to rule breaking. Narcissism tends to engage in immoral behaviour when ego or status is threatened, while sadism reflects a preference for transgressive behaviour that is intrinsically rewarding [43, 44]. These distinctions provide a theoretical basis for associating differential patterns of academic misconduct across the traits, as academic dishonesty can be enacted strategically (e.g., contract cheating), reactively (e.g., exam cheating), or for the enjoyment of norm violation (e.g., lying or deception).

Dark personality traits also differ in their sensitivity to punishment and institutional deterrence mechanisms, which has implications for the frequency and form of academic misconduct. Psychopathy is associated with reduced punishment sensitivity and heightened reward salience [45, 46], suggesting that rule violations may persist even when sanctions are salient. Machiavellianism, by contrast, strategic calculation, deception, and punishment avoidance, suggesting that misconduct may be more likely when perceived benefits outweigh controllable risks [42]. Narcissism may be deterred more by ego-relevant punishment, such as reputational damage or evaluative scrutiny, than by formal sanctions [47]. Sadism is unique in that transgressive behaviour may be intrinsically rewarding, reducing the motivational impact of punishment altogether [27]. These differences provide a framework for understanding why certain forms of misconduct (e.g., contract cheating versus lying) may be differentially associated with distinct dark traits.

### Academic misconduct and dishonesty

Academic misconduct refers to behaviours that violate institutional or scholarly norms of integrity, honesty, and responsibility in educational contexts [48]. In the present review, academic misconduct (or ‘general academic misconduct’) is treated as a broad umbrella construct encompassing dishonest, deceptive, or unethical behaviours undertaken to obtain an unfair academic advantage [49]. This hypernym includes both general ‘dishonesty’ (e.g., lying, misrepresentation, and deceptive practices) and more specific behavioural expressions such as cheating, plagiarism, contract cheating, fabrication and falsification, collusion, and AI-assisted misconduct. Academic misconduct is used as the primary umbrella term, as it subsumes related constructs such as cheating, dishonesty, and academic fraud, which are often treated as conceptually overlapping in the literature. This structure aligns the breadth of the search strategy with the behavioural specificity observed in the empirical literature. These behaviours undermine the fairness, credibility, and value of academic qualifications and are considered serious breaches of academic ethics [50]. Universities and educational institutions define academic misconduct as any attempt to gain an unfair academic advantage through dishonest or deceptive means [49]. Academic misconduct can encompass a broad range of behaviours including cheating or exam cheating, the usage of prohibited electronic devices, unauthorised materials or accessing questions in advance [51, 52]. Plagiarism, defined as presenting someone else’s work, ideas, or words as one’s own without proper acknowledgment, can range from direct copying and pasting to more subtle forms such as paraphrasing without citation [53]. Contract cheating, which is a form of outsourcing academic work, in which students pay or otherwise induce third parties to complete assignments or exams on their behalf and is particularly insidious because it is difficult to detect and undermines authentic assessment [54]. Similarly, collusion is unauthorised collaboration between students on assignments meant to be completed individually and although less overt than cheating or plagiarism, collusion can still violate institutional rules [55]. Lastly, fabrication or falsification is manipulating or inventing data, references, or findings in research or coursework [56]. These actions erode trust in academic output and are especially damaging in scientific contexts [56, 57]. Cheating itself can be enacted through several modes that vary in intentionality and complexity, including the use of unauthorised materials, accessing answers in advance, colluding with peers, impersonation, or receiving real-time assistance during assessments [49, 58–60]. Plagiarism also encompasses multiple forms, such as direct copying, mosaic plagiarism, paraphrasing without attribution, idea plagiarism, and self-plagiarism [61, 62]. Fabrication and falsification may involve either the invention of academic content (e.g., data, citations, quotations), or the manipulation of existing materials through selective reporting, alteration, or omission [63]. These distinctions are relevant for academic integrity research as different forms of misconduct vary in planning, interpersonal involvement, and perceived risk, which may in turn relate to individual difference factors such as personality traits. Such distinctions are theoretically useful because strategic, low visibility, or outsourced forms of cheating (e.g., contract cheating) may be more strongly associated with calculated or instrumental traits (e.g., Machiavellianism [42, 64]), whereas opportunistic or impulsive forms (e.g., exam cheating) may be more closely linked to psychopathy [65, 66] or narcissistic self-enhancement motives [47].

Academic misconduct can also be meaningfully categorised by its motivational and procedural dimensions. Academic integrity literature distinguishes between (a) deception-based misconduct (e.g., lying, falsification, and misrepresentation), (b) knowledge misappropriation (e.g., plagiarism and collusion), (c) assessment cheating (e.g., exam cheating, unauthorized materials, or advance access to questions), and (d) outsourcing or third-party cheating, including contract cheating and, more recently, AI-assisted cheating [51, 58, 67–69]. These categories differ in the degree of planning, interpersonal involvement, technological mediation, and risk tolerance required. Such distinctions are theoretically useful because they support expectations regarding which forms of misconduct may be more strongly associated with particular dark personality traits. For instance, outsourcing and deception-based misconduct often require strategic manipulation, whereas assessment cheating may involve impulsivity or risk-taking. The rise of generative AI tools, such as ChatGPT, has introduced new avenues for academic misconduct due to its ease of use. AI can be used to generate essays, paraphrase a text, or to answer exam questions in real time, which often bypasses plagiarism detection software [69, 70]. These tools blur the line between acceptable assistance and dishonest outsourcing which leads to questions about originality, authorship, and academic integrity [71]. The increasing use of generative AI tools adds an additional layer to this taxonomy by blurring the boundary between legitimate assistance and dishonest outsourcing. Recent advances in generative artificial intelligence tools have introduced new opportunities for technologically mediated academic misconduct, including outsourcing, paraphrasing, automated text generation, and real-time problem solving [69]. Although empirical research is still emerging, theoretical accounts suggest that dark personality traits may differentially relate to AI-assisted misconduct [72] consistent with their motivational profiles. For example, Machiavellianism, characterised by its calculated goal pursuit and strategic exploitation may exploit AI tools instrumentally for strategic or efficiency-based cheating seeing it as low risk and efficient [2, 9]. Narcissism, particularly its grandiose facet, is linked to entitlement and performance orientated self enhancement and may use AI as a convenient and accessible way to maintain self-enhancement in academic performance [47, 73]. Psychopathy, marked by impulsivity and reduced punishment sensitivity [46, 65, 66], may engage in norm-violating uses of AI with minimal concern for institutional sanctions. Everyday sadism, reflecting enjoyment of transgression and cruelty, may also be relevant insofar as AI affords opportunities to transgress rules for intrinsic enjoyment and reward of norm violation. These theoretically derived expectations align with contemporary academic integrity frameworks and provide a basis for interpreting emerging empirical findings.

Motivations for academic misconduct are complex and shaped by various factors. These include fear of failure, high expectations, and a lack of preparedness, along with the perception that there is a low likelihood of being caught and that cheating is socially acceptable among peers [51, 74, 75]. Although situational and institutional factors contribute to academic misconduct, there is increasing evidence that individual personality traits also play an important role in shaping ethical decision making in academic settings [76]. Traits associated with moral disengagement, entitlement, impulsivity, strategic manipulation, low conscientiousness, and dark personality features (e.g., narcissism, psychopathy) have been implicated in dishonest academic behaviours [77]. Emerging research has expanded this focus to include everyday sadism and Machiavellianism, both of which are associated with opportunistic and manipulative rule-breaking [27, 78]. Due to these personality factors and the influence on cheating behaviour in an academic setting, a review of the cluster of dark traits known as the Dark Tetrad is warranted.

There is a substantial body of research that has examined individual Dark Tetrad traits in relation to academic misconduct independently of the full Dark Triad or Tetrad framework. For example, psychopathy (and particularly primary psychopathy) has been linked to academic dishonesty and academic cheating behaviours [79, 80], with further associations of outsourcing academic work to AI systems [81]. Machiavellianism has similarly been associated with academic cheating [82], with its amoral manipulation and strategic exploitation aspects identified as key associations of academic cheating behaviour [83]. Narcissism has been linked to academic dishonesty and cheating, particularly through its power and exhibitionism dimensions [84], whereas sadism has received comparatively little attention in single trait academic context studies despite its association with intrinsic rule breaking and norm violation [27]. These trait specific literatures provide important conceptual foundations for understanding the Dark Tetrad traits and academic misconduct. However, because Dark Tetrad traits frequently show high overlap, intercorrelation, and share behavioural variance representing a broader ‘dark core’ [85–87], studies examining these traits in isolation limits the ability to determine their relative and potentially overlapping contributions. The present review therefore focuses specifically on studies measuring the complete Dark Triad or when available the full Dark Tetrad, allowing direct comparison of shared and unique associations of the Dark Tetrad with academic misconduct and cheating.

### Academic integrity

Academic integrity is commonly conceptualised as the positive counterpart to academic misconduct, referring to the shared institutional values and practices that promote honesty, trust, fairness, respect, responsibility, and accountability in educational contexts [88, 89]. Literature on academic integrity emphasises that misconduct does not arise solely from individual dispositions, but from the interaction between personal characteristics and contextual factors, including assessment design, peer norms, institutional culture, and perceptions of enforcement [50, 51, 90]. Further highlighted is the need to understand both structural drivers (e.g., assessment vulnerability, contract cheating markets, technological affordances) and individual level associations that may increase susceptibility to rule violations [91, 92]. Within this broader integrity framework, personality differences remain theoretically important, as students may vary in their sensitivity to punishment, moral disengagement tendencies, strategic reasoning, and willingness to exploit available opportunities. Situating the Dark Tetrad within an academic integrity context therefore allows for a more nuanced understanding of how dispositional risk factors may interact with institutional environments to shape patterns of academic misconduct.

### Aims of the review

This paper presents a systematic review and narrative synthesis that examines the existing evidence on the relationship between Dark Tetrad personality traits and academic misconduct, including behaviours such as cheating and plagiarism. Given the growing interest in dark personality research and the rapid rise in the use of generative AI in academic contexts, such a review is both timely and necessary. By examining this relationship, the review aims to determine whether individual differences in Dark Tetrad traits are associated with academic misconduct behaviours. More specifically, it seeks to clarify how these traits are associated with academic dishonesty, and to contribute to a deeper understanding of the psychological correlates of such behaviour, potentially informing strategies to address academic misconduct in the future. The literature on the dark traits in academic contexts remains fragmented, with studies varying considerably in how both the Dark Tetrad traits (e.g., Short Dark Triad, Dirty Dozen, Short Sadistic Impulse Scale) and academic misconduct (e.g., cheating, plagiarism, dishonesty, contract cheating, and more recently AI-assisted misconduct) are operationalised, making it difficult to determine the consistency and specificity of observed associations. Furthermore, while individual studies often report positive associations between the dark traits and academic misconduct, findings are not uniform across traits and behaviours, and no prior synthesis has examined whether including sadism alongside the Dark Triad offers additional conceptual insight into academic conduct. This distinction is in line with the central aim of the synthesis, to examine whether the Dark Triad/Tetrad relate to academic misconduct as a coordinated cluster when traits are measured concurrently, rather than to provide an exhaustive review of isolated single trait effects. Therefore, A key objective of this review was to evaluate whether Dark Tetrad traits demonstrate differential or convergent associations with academic misconduct when measured concurrently within the same study designs. To our knowledge, no systematic review has synthesised this evidence, leaving unclear which dark traits are most reliably associated with academic misconduct and whether the complete Dark Tetrad offers a conceptually useful, though currently preliminary, framework for understanding personality correlates of academic misconduct related outcomes. This review was conducted in line with PRISMA guidelines and includes a narrative synthesis of findings across studies rather than a quantitative meta-analysis. Furthermore, this review represents the first narrative synthesis exploring all Dark Tetrad traits in relation to academic misconduct.

## Method

This current systematic review and narrative synthesis was guided by the Cochrane Method and presents the search methods and findings according to the well-established Preferred Reporting Items for Systematic Reviews and Meta-Analyses (PRISMA) guidelines [93, 94]. Below is the protocol used to conduct this review.

### Eligibility criteria

Studies were included in this systematic review and narrative synthesis if they met the following criteria: (1) they reported empirical, peer-reviewed research on the Dark Triad or Dark Tetrad traits; (2) they measured an outcome related to academic dishonesty (e.g., plagiarism, cheating, or other forms of academic misconduct); and (3) they were published in English between 2010 and 2025. Studies published from 2010 onward were included to align with the emergence of the Dark Triad/Tetrad construct and the availability of validated measures for these traits. This time frame ensured conceptual consistency and methodological comparability across included studies. Studies that assessed only one or two dark traits in isolation were excluded as a deliberate scope boundary rather than because such studies lack value. This helped to ensure consistent construct representation and to enable meaningful comparisons of the unique and shared variance among the traits. The primary aim of this review was to synthesise studies in which the Dark Triad/Tetrad were measured together, allowing patterns across traits to be interpreted within the same empirical context. This criterion preserved construct comparability and supported the reviews focus on dark personality clusters rather than isolated trait effects. However, it does also narrow the evidence base and likely excluded relevant single trait studies, including work on psychopathy subtypes, narcissism facets, and everyday sadism. Consequently, the conclusions of this review should be interpreted as applying to co-measured Dark Triad/Tetrad evidence rather than to the full body of research on each individual dark trait. This criterion was also intended to minimise distortions arising from heterogeneous measurement approaches across traits.

Given the conceptual heterogeneity of academic misconduct outcomes, the methodological diversity in the operationalisation of dark traits (e.g., SD3, DTDD, Dirty Dozen, NPI, SRP), and the variability in statistical reporting formats (e.g., correlations, standardized betas, odds ratios, path coefficients, and mediation models), a meta-analytic synthesis was not appropriate. These factors preclude the assumption of construct and metric commensurability required for effect size pooling [95, 96]. We considered whether a narrower subset of studies could support partial quantitative synthesis, however, even the largest outcome clusters such as cheating and plagiarism, varied substantially in outcome type (e.g., attitudes, intentions, self-reported behaviour), trait measurement, covariate adjustment, and reported effect metrics. Other categories such as contract cheating, AI assisted misconduct, fabrication/falsification, and sadism specific analyses contained too few comparable studies to support meaningful pooling. This approach was appropriate for the present systematic and narrative synthesis review, which aimed to synthesise evidence on the Dark Tetrad as an integrated personality framework rather than aggregate effects of individual traits as in a meta-analysis and is consistent with prior syntheses examining dark personality traits in related behavioural domains which similarly excluded studies assessing only a subset of traits [96, 97]. Additionally, this review focused specifically on subclinical levels of the Dark Tetrad traits, rather than clinical diagnoses, in order to enhance the generalisability of findings and deepen understanding of personality traits as they manifest in everyday contexts [98].

Studies were not included in this review based on the following criteria: (1) the study did not report all members of the Triad or Tetrad (2) the dishonesty or misconduct was not related to education or academic setting and (3) the article did not present any data or was ‘grey’ literature (e.g. theses, book chapters, review articles etc.).

This review followed a systematic search and screening process consistent with PRISMA. Due to heterogeneity in study designs, outcome measures, operationalisations of academic misconduct, and personality measurement (e.g., SD3, Dirty Dozen, Short Dark Tetrad), a quantitative meta-analysis was not feasible. We therefore adopted a narrative synthesis approach to describe and integrate the findings across studies.

### Search strategy and data repositories

The databases PubMed, PsycINFO, and Google Scholar were searched in August and September 2025 using a search strategy based on two concepts- Dark Tetrad and academic misconduct. The terms that were searched for in the title and abstracts can be found in Table 1. Broader search terms (e.g., dishonesty, misconduct) were used to ensure sensitivity, while narrower behavioural categories were applied during data extraction and synthesis. PsycINFO was selected as the primary disciplinary database due to its comprehensive coverage of psychological and behavioural science literature, including research on dark personality traits. PubMed captured interdisciplinary and clinical publications, and Google Scholar was used as a supplementary search tool capturing grey literature and interdisciplinary publications not consistently indexed in traditional bibliographic databases. Due to the high volume of results, ranking instability, and lack of advanced filtering options in Google Scholar, only the first 200 results (sorted by relevance) were screened, consistent with best practice recommendations [99–101]. Results are often rank ordered by relevance and lower ranked records have been shown to decline in relevance with diminishing returns. This allows for greater transparency and feasibility while reducing the risk of omitting non-indexed material. While this approach improved feasibility and provided supplementary coverage, it can limit reproducibility because Google Scholar rankings can vary over time and by search context. This may have also resulted in relevant lower order records being missed. Accordingly, Google Scholar findings were treated as supplementary rather than equivalent to database indexed records. Following reviewer recommendations, a supplementary search was conducted on January 10th, 2026, in Scopus and ERIC databases using the identical search syntax. Web of Science could not be accessed due to institutional licensing restrictions and was therefore not used. This does represent a limitation of the current review, as Web of Science may index records not captured in PubMed, PsycINFO, Scopus, ERIC, or Google Scholar. However, Scopus was included as a broad multidisciplinary database with substantial overlap with Web of Science, and the supplementary Scopus/ERIC search identified three additional eligible studies. This process strengthened coverage beyond the original search but does not eliminate the possibility that some relevant studies were missed. Full text screening revealed three additional eligible studies beyond those identified in the original search, consistent with recommendations for completeness checks in systematic reviews and previous literature [95, 102, 103]. This approach aligns with PRISMA recommendations for documenting database specific search procedures and screening boundaries.

**Table 1:** Search Terms Utilised in the Formal Search

Academic Misconduct	Dark Tetrad
Academic Dishonesty	Narcissis*
Academic Cheating	Machiavellian
Cheating	Psychopath
Plagiarism	Sadis*
Test Cheating	Dark Triad
Exam Cheating	Dark Tetrad
Contract Cheating	
Falsification	
Academic Integrity	
Generative AI	

Search terms with an asterisk * indicate database truncation to capture multiple word stems (e.g., sadis retrieves sadism, sadistic, and related terms)

### Study selection

**Fig. 1:**
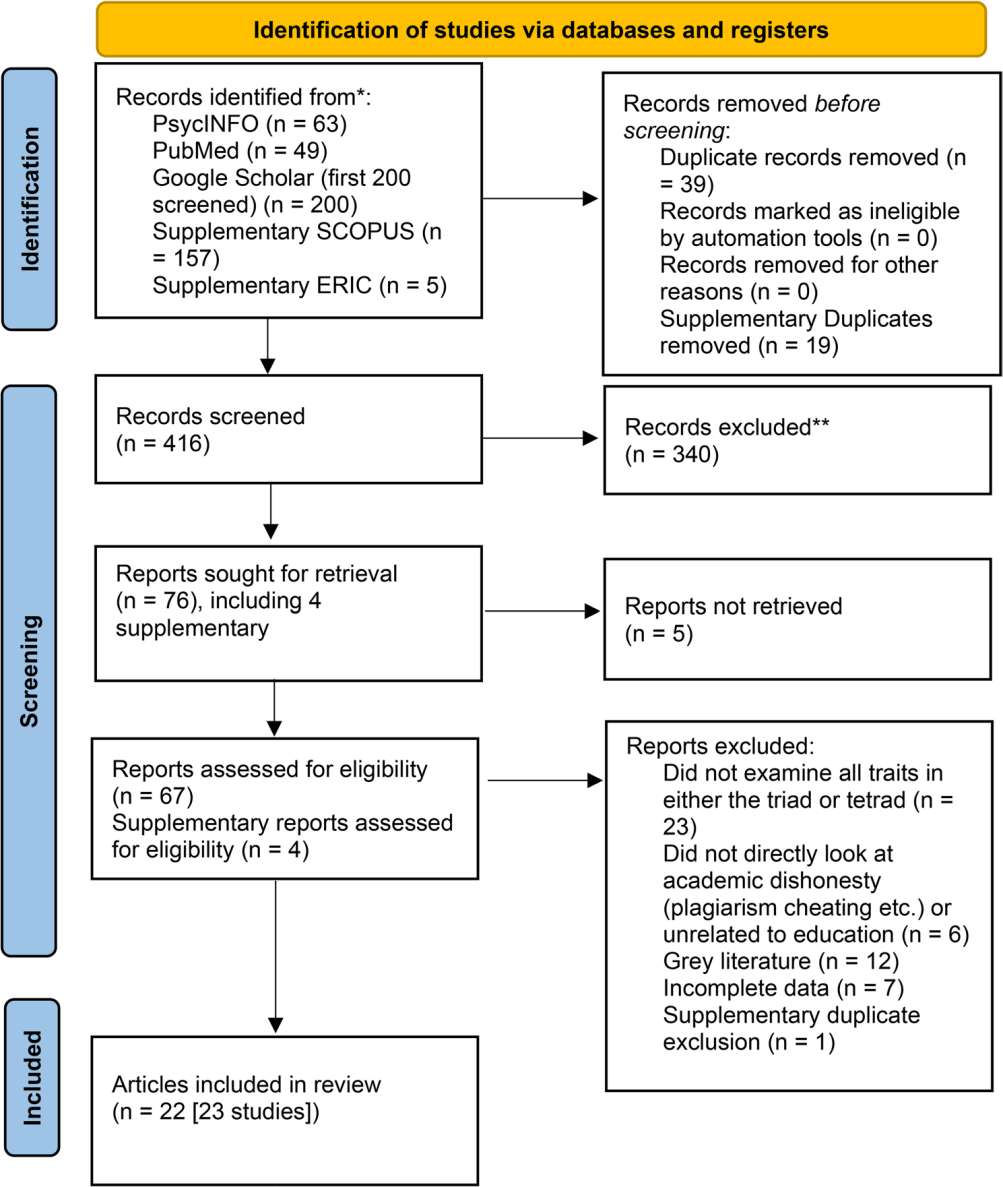
PRISMA Flow chart depicting the study selection process

The search strategy described above was applied to each database, and all identified records were imported into a single EndNote library. Duplicate articles found across multiple databases were removed, followed by screening of titles and abstracts. Articles deemed ineligible during this initial screening were excluded. Full texts of the remaining eligible articles were then retrieved and assessed against the exclusion criteria. Articles that did not meet the criteria were removed, and the remaining studies were included in the final systematic review and narrative synthesis. Screening and selection were conducted independently and in duplicate by two reviewers to ensure methodological rigour. Inter-rater agreement was calculated for original search for categorical decisions at full text screening stage using Cohen’s κ = 0.73, which indicated substantial reliability [104], and any discrepancies were resolved through discussion and consensus. The three additional full-text articles identified in the supplementary Scopus/ERIC search were also screened independently by both reviewers, with 100% agreement on their inclusion. This dual-review process minimised potential bias and ensured transparency in study inclusion decisions. Numerical data (e.g., correlation coefficients and regression parameters) were extracted by one reviewer and checked for accuracy by a second reviewer. Inter-rater reliability statistics were not calculated for these continuous values because the review employed a narrative rather than quantitative meta-analytic synthesis. See Fig. 1 for the detailed study selection process.

### Data collection and quality assessment

A data extraction table was developed to compile relevant information from each eligible study. This table captured key article characteristics (authors, year, country), sample details (age, gender), measurement methods for the personality traits, types of academic misconduct examined (e.g., cheating, plagiarism), main findings (including univariate and multivariate statistical results), noted limitations, and the overall quality rating assigned to each study. Studies were grouped by type of misconduct (e.g., cheating, plagiarism, contract cheating, dishonesty, falsification/fabrication, AI-assisted misconduct) and by trait (narcissism, Machiavellianism, psychopathy, sadism). We synthesised results narratively within and across these groups to identify patterns of association and examine which traits were most consistently linked with specific forms of misconduct. This constitutes a narrative synthesis embedded within a systematic review framework.

This review used the AXIS tool to assess the quality of all the eligible studies for this review and was designed for use with cross sectional and observational studies [105]. The AXIS tool contains 20 items that requires a yes/ no, or don’t know (1 = Yes, 2 = No/Don’t Know) to questions such as ‘Was the study design appropriate for the stated aim(s)?’ that helps ascertain an articles quality. A quality score out of 20 is then generated from these responses. It is worth noting that the interpretation of the scores guided by the AXIS tool is still subjective, thus certain thresholds of scores are followed as a guideline (1–7 = low quality; 8–14 = medium quality; 15–20 = high quality). The overall quality score can be found in Additional File 1, the scores for each individual component of the eligible articles can be found in Additional File 2: Table S1 and Table S2 (Also available on Open Science Framework https://osf.io/46ng2).

## Results

### Study selection

The initial search yielded a total of 273 unique articles, after the supplementary search this increased to 416 unique articles. Once the titles and abstracts were screened, including the supplementary search, 71 articles were assessed for eligibility after full text screening. In total, 22 articles comprising 23 eligible studies were included. One article contained three studies^2^; two were eligible and included, while one was excluded at full-text screening (full details are presented in Fig. 1.). Inter-rater agreement between reviewers at original full text pool was substantial (κ = 0.73).

### Study characteristics

All 22 articles were published between 2010 and 2025, with 16 being published from 2021 onwards indicating a recent interest in dark personality trait research. All studies used a mixed gender and community sample with majority targeting undergraduate university students (*n* = 21 studies) although there were samples targeting community (*n* = 1), as well as undergraduate and graduate combined samples (*n* = 1). Specific educational year was inconsistently reported across studies and therefore could not be systematically extracted. Where reported, the average age across all studies was 23.38 years. Samples sizes from across studies ranged from *N* = 107 to *N* = 1201. The Triad of traits (*n* = 19) was explored far more than the Tetrad (*n* = 3). There was 12 unique different measures to assess the dark traits, the Short Dark Triad (SD3; [107]) was the most utilised (*n* = 12) followed by the Dirty Dozen (*n* = 6) (DTDD; [108]) which both capture the dark traits in a single questionnaire. Most included studies operationalised psychopathy as a composite Dark Triad/Tetrad score rather than distinguishing primary, secondary, or facet level psychopathy. Consequently, the synthesis reports psychopathy at the level used by the primary studies, while noting subtype specific findings where available. The remainder of the measures aimed to capture a single trait of the Triad or Tetrad so were administered in combination with each other. Only a minority of studies had controls for social desirability bias (*n* = 2). See Additional File 1 for the synthesised table of the included studies that encompasses detailed mean age, gender split, and sample size for each sample.

### Narrative synthesis of major findings

#### Dishonesty

Four studies examined dishonesty. Baughman, Jonason [109] and Forsyth, Anglim [110] both found that the dark traits were positively associated with lying frequency, lying propensity, and positive emotions when lying. Baughman, Jonason [109] reported that Machiavellianism (*β* = 0.18) and psychopathy (*β* = 0.10) were positively associated with greater effort invested in lying, while narcissism (*β* = 0.14) uniquely was positively associated with perceived believability of lies. Forsyth, Anglim [110] additionally found all Tetrad traits to be negatively correlated with cognitive load and negative emotions when lying, with Machiavellianism (*β* = 0.31) and sadism (*β* = 0.12) showing significant positive associations of lying in an academic context. Lingán-Huamán, Dominguez-Lara [111] reported direct effects of Machiavellianism on cheating (male *β* = 0.65; female *β* = 0.69), plagiarism (male *β* = 0.19; female *β* = 0.21), and falsification (male *β* = 0.23; female *β* = 0.60), and of psychopathy on plagiarism (male *β* = 0.41; female *β* = 0.26). Koscielniak, Enko [112] found Machiavellianism, narcissism, and psychopathy to all positively correlate with dishonesty on an individual level, whereas only Machiavellianism was positively correlated on a collective level.

#### Cheating

Ten studies focused on cheating. Williams et al. ([106], Study 1), Zhang, Paulhus [113], Cheung and Egan [114], and Kokkinos and Antoniadou [115] consistently found positive correlations between the Dark Triad traits and cheating, with psychopathy showing the strongest association (e.g., Williams et al., *β* = 0.50; Zhang et al., *β* = 0.40). Esteves et al. [116] reported that Machiavellianism (*β* = 0.28) and narcissism (*β* = 0.16) were positively associated with engaging others in cheating (*F* (4.30) = 22.72, *p* < .001, *R²* =0.17) and initiating cheating (*R²* =0.15, *F* (14, 951) = 19.97, *p* < .001). He, Zheng [117] found Machiavellianism and psychopathy were positively associated with cheating, with effects mediated by performance avoidance (indirect effect = 0.06). Curtis [118] also observed positive associations for all three traits with cheating and plagiarism. Turnipseed and Landay [119] found only Machiavellianism (*β* = 0.03) positively associated with academic cheating appropriateness with psychopathy and narcissism non-significant. Clemente, Espinosa [120] found positive correlations between all four dark traits and cheating but only found Machiavellianism (*β* = 0.20) positively associated with cheating whereas sadism (*β* = − 0.15) showed a negative association. In the context of exam cheating, Mungall, Fazaa [121] found that two Machiavellianism factors showed opposing associations, one positively (Planfulness- LnOR 0.39) and one negatively associated (Agency- LnOR − 0.41) with past exam cheating behaviour. Vulnerable narcissism (LnOR 0.41 ) and the interpersonal manipulation (LnOR 0.45) psychopathy factor were also positively associated with past exam cheating behaviour. Further regressions found grandiose narcissism (*β* = 0.11) to have a positive association and the psychopathy factor Affective (*β* = − 0.27) a negative association of endorsing cheating behaviour.

#### Plagiarism

Five studies reported on plagiarism. Williams et al. ([106], Study 2), Curtis, Correia [122], Curtis [118], and Kokkinos and Antoniadou [115] each found positive associations between the dark traits and plagiarism, with Curtis, Correia [122] reporting that these effects were fully mediated by academic entitlement. Ternes, Babin [123] found that psychopathy, particularly primary psychopathy, was most strongly associated with plagiarism (*r* = .22; *β* = 0.22), alongside other forms of misconduct, whereas secondary psychopathy and Machiavellianism had weaker or inconsistent associations.

#### Fabrication and falsification

Two studies examined falsification and fabrication. Ternes, Babin [123] reported positive associations between psychopathy and fabrication (*r* = .12) and falsification (*β* = 0.33), with primary psychopathy showing the strongest effects after controlling for impulsivity. Lingán-Huamán, Dominguez-Lara [111] found that psychopathy was positively associated with falsification for males (*β* = 0.39), while Machiavellianism was positively associated with falsification for both sexes (male *β* = 0.23; female *β* = 0.60).

#### General academic misconduct

Three studies examined general academic misconduct. Ternes, Babin [123] found significant associations across multiple misconduct variables, with primary psychopathy showing the most consistent pattern of positive associations. Veríssimo, Conrado [124] reported that all three dark traits correlated with misconduct but only Machiavellianism (*β* = 0.28) remained significant when controlling for covariates such as academic year and severity of penalty. Stojanov, Hannawa [125] also reported positive associations with all three Dark Triad traits, with the SACCIA framework communication style moderating the strength of effects for psychopathy and narcissism.

#### Contract cheating and collusion

Contract cheating and collusion were examined in two studies. Rundle, Curtis [126] found Machiavellianism showed the strongest positive association of not engaging in contract cheating due to fear of detection (*β* = 0.42), self-efficacy and mistrust of others (*β* = 0.21), lack of opportunity (*β* = 0.10), and barriers to consideration (*β* = 0.25). Psychopathy was negatively related to morals and norms (*β* = –0.16), while narcissism showed no significant effects. Kokkinos and Antoniadou [115] reported Machiavellianism and psychopathy to be positively associated with unauthorised collaboration, with psychopathy’s effect moderated by moral disengagement (*b* = − 0.12, *p* < .05).

#### Academic fraud

One study investigated academic fraud^3^. Srirejeki, Faturokhman [127] reported that the Dark Triad had significant positive associations with academic fraud intentions (*β* = 0.35), with Machiavellianism (*β* = 0.12), narcissism (*β* = 0.10), and psychopathy (*β* = 0.20) each exerting positive effects.

#### Use of AI to cheat

Two studies examined AI-assisted cheating. Greitemeyer and Kastenmüller [128] found all three dark traits positively correlated with intention to use ChatGPT, though only narcissism (*β* = 0.18) and psychopathy (*β* = 0.21) remained significant when HEXACO traits and chatbot quality were controlled *F*(9, 282) = 4.64, *p* < .001, *R²* = 0.13. Sun, Tang [129] similarly found narcissism (*β* = 0.20), psychopathy (*β* = 0.19), and sadism (*β* = 0.18) had positive associations with generative AI misconduct, while Machiavellianism was not significant.

## Discussion

This systematic review and narrative synthesis aimed to review the available evidence examining the Dark Triad and Tetrad traits with academic misconduct such as cheating and dishonesty. The synthesised evidence indicates that Machiavellianism and psychopathy were the traits more consistently associated with academic misconduct across the included studies than narcissism, although these comparisons should be interpreted cautiously given variation in trait measures, misconduct outcomes, and statistical models. Everyday sadism, examined in a smaller number of studies, showed some associations with dishonest behaviour in academic contexts, but the evidence base remains sparse for strong conclusions and warrants cautious interpretation. Overall, these findings were observed across diverse cultural contexts and multiple forms of misconduct, including cheating, plagiarism, contract cheating, dishonesty, falsification, fraud, and the use of artificial intelligence for academic purposes. When interpreted through the misconduct taxonomy introduced earlier (i.e., deception-based misconduct, knowledge misappropriation, assessment cheating, and outsourcing based misconduct), clearer patterns appear. The included studies primarily assessed broad categories such as cheating, plagiarism, dishonesty, contract cheating, and AI-assisted misconduct, rather than fine grained subtypes. Accordingly, the synthesis focuses on these operationalised categories while interpreting them within the broader motivational and procedural framework outlined in the Background.

Importantly, these cross study patterns should be interpreted cautiously as the included studies did not always measure equivalent constructs. Dark traits were assessed using different instruments, including brief omnibus measures and separate trait specific scales. Academic misconduct too was operationalised variously as attitudes, intentions, self-reported past behaviour, general dishonesty, plagiarism, cheating, contract cheating, or AI assisted misconduct. The statistical models also varied across studies, including correlations, regression coefficients, odds ratios, and mediation models. Therefore, comparisons across traits and misconduct categories should be treated as indicative patterns within a heterogeneous evidence base rather than direct like for like comparisons. Furthermore, these patterns should also be interpreted in light of uneven outcome coverage, as cheating and plagiarism were represented more frequently than contract cheating, fabrication/falsification, academic fraud, and AI-assisted misconduct. In the following sections, empirical findings from the reviewed studies are distinguished from theoretical interpretation drawn from the established Dark Triad/Tetrad trait models.

### Narrative synthesis of major findings

Across the included studies, psychopathy showed the most consistent pattern of positive associations with academic misconduct outcomes, including cheating, plagiarism, contract cheating attitudes, dishonesty, and AI-assisted misconduct. This pattern can be interpreted in light of established theoretical accounts of psychopathy, which emphasise the impulsive, callous, and thrill-seeking characteristic nature of psychopathy [2]. From this interpretation, the impulsive and thrill-seeking aspects of psychopathy could explain cheating in exam contexts, where immediate gratification is prioritised over long-term consequences. Similarly, the callous disregard for rules or fairness may foster plagiarism or contract cheating, where the individual benefits at the expense of institutional integrity. In newer domains, such as AI-assisted misconduct, psychopathy may facilitate rule-breaking simply because the behaviour is available and low-effort, with little concern for repercussions. Similarly, this may also appeal to Machiavellianism, where low effort opportunities for undetected exploitation align with strategic goal pursuit, and to narcissism, where AI tools can serve self enhancement motives or performance optimisation. This suggests that emergent academic technologies may reveal convergent behavioural patterns across the dark traits, albeit for distinct psychological reasons. Moreover, this pattern of misconduct is also consistent with evolutionary accounts that conceptualise psychopathy as having a fast life history, prioritising immediate gratification, cheating strategies, and social exploitation in cooperative systems [39–41]. Although the present review cannot test this mechanism directly.

The absence of guilt or remorse allows individuals high in psychopathy to engage in misconduct without experiencing the moral conflict that might deter others [9]. However, only one study made the distinction between primary and secondary psychopathy and found primary psychopathy, often related to callousness and manipulation, showed the strongest associations on multiple variables of academic misconduct leaving secondary psychopathy, related to impulsivity and emotional dysregulation, not significant. This pattern may suggest that, in the available studies, academic dishonesty may not be uniformly linked to reactive or impulsive tendencies and may reflect the calculated, unemotional exploitation of opportunities. However, because only one study distinguished psychopathy subtypes, this interpretation remains tentative. Thus while the present synthesis often refers to psychopathy as a composite trait, this reflects the way psychopathy was operationalised in most included studies rather than an assumption that psychopathy is theoretically unitary. Where subtype or facet level data were available, they suggested potentially meaningful distinctions, particularly stronger links for primary psychopathy and callous-interpersonal features than for secondary impulsive features. Future research should build on this distinction by systematically examining both subtypes of psychopathy across academic contexts, as this could clarify whether misconduct is primarily driven by cold, strategic traits rather than by emotional instability.

Machiavellianism also showed relatively consistent associations with calculated and manipulative forms of misconduct, such as cheating and plagiarism, but was not associated with contract cheating. The strength and direction of effects also varied across measures and models. These associations may be interpreted in light of theoretical accounts of Machiavellianism defined by strategic deception, long-term planning, and instrumental exploitation of others [20]. Such behaviours require forethought, manipulation of systems, and sometimes coordination with third parties (e.g., essay mills). From this perspective, some forms of academic misconduct may be attractive because they allow individuals to obtain academic advantage through planning, concealment, or manipulation of assessment systems. Machiavellians may perceive these behaviours as adaptive strategies for academic success, reflecting their broader orientation toward manipulative goal pursuit. Even in more straightforward behaviours like plagiarism, Machiavellianism may motivate calculated risks, where the perceived benefits outweigh the likelihood of detection. Evidence suggests that Machiavellians may avoid contract cheating, not because of moral concerns, but due to pragmatic considerations such as fear of detection, mistrust of third-party providers, or perceived lack of opportunity [126]. One possible interpretation is that Machiavellianism may be linked to calculated forms of academic dishonesty, it simultaneously restrains behaviours that involve high risk and reliance on external parties and outsourcing academic work where control is relinquished to others such as generative AI. However, it is theoretically noteworthy that Machiavellianism was not associated with contract cheating, given that contract cheating possesses features typically associated with Machiavellian strategy (e.g., premeditation, outsourcing, and risk-reward calculation). From a motivational standpoint, one might expect Machiavellianism to favour such behaviours, as suggested by work linking Machiavellianism to calculated rule breaking and exploitative tactics in other domains [9, 42]. One possible explanation is that contract cheating involves reputational and detection risks that are not easily controlled by the perpetrator, whereas plagiarism and exam cheating may afford greater tactical control. Alternatively, the null effect may reflect contextual factors (e.g., cultural norms, sanctions, or institutional detection systems) rather than dispositional ones. Future work incorporating gradations of misconduct based on detectability, risk, and payoff would help clarify whether Machiavellianism differentiates between forms of academic misconduct.

Narcissism showed a weaker and more context dependent pattern of association with academic misconduct than psychopathy and Machiavellianism, though some studies reported significant links with cheating, plagiarism, dishonesty, and AI-assisted cheating. The defining features of narcissism, grandiosity, entitlement, and a need for admiration, may help explain its relationship with certain forms of misconduct [7]. This pattern may be interpreted through the distinction between grandiose and vulnerable narcissism. Grandiose narcissism, characterised by overt self-importance and dominance, may be relevant when misconduct serves self-enhancement and status maintenance, for example to secure high grades that affirm superiority. Vulnerable narcissism, by contrast, reflects hypersensitivity to criticism and fragile self-esteem, and may be relevant to when misconduct functions as a defensive response to fear of failure or anticipated negative evaluation. Consistent with this, one study in the present review [121] found vulnerable narcissism was associated with past exam cheating behaviour, whereas grandiose narcissism was more strongly associated with favourable attitudes towards cheating, suggesting that different facets of narcissism may be implicated in behaviour versus attitudinal endorsement of misconduct. Narcissistic individuals often strive to maintain a positive self-image and protect their fragile self-esteem, which can manifest as cheating to avoid failure or to secure achievements that bolster their status [130]. In academic contexts, plagiarism or exam cheating may provide a route to maintain superiority and demonstrate competence, even if these accomplishments are unearned. Plagiarism or AI-assisted writing tools may be attractive as a way to secure recognition for polished work, even if the underlying ability is lacking.

Narcissism, however, does not appear to consistently be associated with more strategic or calculated forms of misconduct, such as contract cheating, nor is it as robustly linked to dishonest behaviours as Machiavellianism or psychopathy is. This may reflect the motivational nature of narcissism: rather than being driven by cold calculation or impulsive callousness, narcissistic misconduct seems to be situational, emerging primarily when opportunities arise to enhance one’s reputation or protect against ego threats. The weaker and more context-dependent pattern of narcissism related findings suggests it plays a secondary role in academic dishonesty, shaped by self-enhancement motives rather than a stable orientation toward exploitation. Future research should explicitly distinguish grandiose and vulnerable narcissism when examining academic misconduct as treating narcissism as unidimensional may obscure subtype specific effects.

Sadism was examined in fewer studies than the other dark traits, and the available evidence is preliminary and should therefore be interpreted cautiously. Some included studies linked sadism with academic dishonesty, including lying in academic contexts and AI-assisted cheating, however, there was a small number of studies that may limit confidence in the consistency and generalisability of these associations. Theoretically, sadism differs from the other dark traits in that it reflects a direct motivation to derive pleasure from causing harm or manipulating others [27]. In academic settings, this may not translate into overt aggression, but rather into a willingness to violate rules and norms simply for the enjoyment of transgression or the empowerment it provides. Forsyth, Anglim [110] for example, found that sadism was associated with lying in academic contexts, aligning with the notion that sadistic individuals experience positive emotions from deception. Sun, Tang [129] reported that sadism was associated with AI-assisted cheating, suggesting that even technologically mediated misconduct can provide opportunities to exercise dominance and disregard for institutional rules.

A possible interpretation is that sadism’s association with academic misconduct may be less about strategic gain as in Machiavellianism, or self-enhancement as in narcissism, and more about the intrinsic satisfaction gained from the act itself. Buckels, Jones [27] argue that sadists engage in antisocial behaviour ‘for fun’ which may make them more willing to cheat even when personal benefit is minimal. In academic contexts, this could involve enjoying the subversion of academic norms or deceiving evaluators, regardless of practical necessity. Clemente, Espinosa [120] reported that all four dark traits correlated with cheating, but only Machiavellianism showed a positive multivariate association whereas sadism was negatively associated with cheating attitudes, suggesting that sadism may sometimes constrain rather than amplify certain forms of misconduct when strategic self-interest is low. Koscielniak, Enko [112] extended the evidence on dishonesty by distinguishing individual versus collective dishonest behaviour, finding that while Machiavellianism, narcissism, and psychopathy were all associated with individual dishonesty, only Machiavellianism was positively associated with dishonest behaviour at the collective level, consistent with its strategic and instrumental orientation. Finally, Mungall, Fazaa [121] provided more fine-grained evidence that specific subcomponents of Machiavellianism, narcissism, and psychopathy differentially were associated with exam cheating behaviour and attitudes, supporting the view that both trait profiles and sub facet structure matter for understanding which forms of academic misconduct are most likely in particular individuals.

In sum, within the limits of a heterogeneous evidence base, psychopathy and Machiavellianism appeared to show the most consistent associations with academic misconduct outcomes, while narcissism showed weaker and more context dependant associations and sadism remains under-researched and evidence should be considered preliminary. Across misconduct types, traits associated with manipulation, callousness, and impulsivity (Machiavellianism and psychopathy) appeared most commonly implicated. The variation in outcome measures and statistical models means these patterns should be interpreted as tentative rather than definitive. Although the breadth of evidence is still limited, these traits appear to be linked with both traditional forms of misconduct (e.g., cheating, plagiarism, fabrication) and more contemporary behaviours (e.g., contract cheating and AI-assisted misconduct). Theoretically, the Dark Tetrad traits may converge on similar misconduct behaviours for different psychological reasons, Machiavellianism through strategy and control, psychopathy through impulsive rule-breaking, narcissism through self-enhancement, and sadism through intrinsic enjoyment of transgression. Mapping academic misconduct along these motivational pathways may prove more informative than treating behaviours as interchangeable. These patterns imply that academic misconduct behaviours may not be interchangeable from the perpetrator’s perspective; rather, their appeal may depend on the behavioural affordances offered by each misconduct type. Variables such as detectability, payoff structure, technological mediation, reputational risk, and required effort may function as situational moderators that differentially attract each trait. For example, contract cheating and plagiarism vary in both detectability and reputational stakes, while AI-assisted cheating offers low effort, ambiguous detectability, and minimal interpersonal engagement. Mapping misconduct along such dimensions may provide a more nuanced account of why certain traits gravitate toward specific behaviours. Developing such a framework would allow future research to model academic misconduct not merely as a unitary construct, but as a behavioural space structured by risk–reward trade-offs and motivational affordances.

### Limitations and future directions

The limitation that appeared most frequently in the articles is the use of self-report questionnaires such as the SD3 and DTDD to capture the traits or assess academic dishonesty therefore introducing a self-report bias. Eighteen of the studies identified self-report bias as a limitation [106, 109–112, 114, 116–119, 121–125, 128, 129]. In studies where the outcome variables involve cheating or dishonest behaviour, typically considered socially undesirable, the dark traits often show a tendency toward social desirability bias, as individuals high in these traits may attempt to present themselves in a more favourable light [131]. Additionally, sadism has been noted for its association with exaggerated or preposterous responses on self-report measures, further complicating the reliability of self-assessments in this context [132]. Only two studies in this review [118, 126] included an instrument to control for social desirability bias, further research should seek to control for social desirability bias and deceptive responding.

Furthermore, the capturing of the dark traits themselves is subject to similar biases and limitations. A majority of the studies in this review used the SD3 which aims to capture Machiavellianism, narcissism, and psychopathy with nine items per trait. This is further reduced to seven items per trait for the SD4 when sadism is measured. Criticism has been levied at these measures for not comprehensively measuring the traits or subtypes which is particularly relevant for psychopathy, treating it as a single composite may obscure differential associations between primary psychopathy’s callous-interpersonal features, secondary psychopathy’s impulsive features, and that the overall trait distinctiveness may vary based on population [133]. Nonsignificant results may therefore potentially be explained by the failure to capture the subtypes [119]. Similarly, the DTDD further reduces the items to four for each Dark Triad trait and faces similar criticism including poor psychometric properties [134]. Future research should consider further refinement of capturing the traits combining the use of self-reports with observer reports [135].

Another limitation concerns the uneven distribution of misconduct outcomes across the included studies. Cheating and plagiarism were examined more frequently than contract cheating, collusion, fabrication/falsification, academic fraud, or AI assisted misconduct. As a result, conclusions are better supported for broad cheating and plagiarism related outcomes than for less frequently studied categories. This imbalance limits the generalisability of trait specific patterns across the full range of academic misconduct behaviours and means that findings for emerging or lower frequency categories, particularly AI assisted misconduct and contract cheating, should be interpreted cautiously. Future research should examine a broader range of misconduct types using comparable measures so that trait misconduct patterns can be evaluated more evenly across categories.

Although every effort was made to maintain methodological transparency, including duplicate screening and quality assessment, a limitation should be acknowledged. Although a quantitative meta-analysis was considered, the substantial heterogeneity in trait measurement (e.g., SD3 vs. Dirty Dozen vs. Short Dark Tetrad), outcome operationalisation (e.g., academic misconduct composites), and statistical reporting (e.g., correlations, regression coefficients, odds ratios, indirect effect, and mediation models) precluded effect size pooling without substantial transformation assumptions. We also considered whether partial quantitative synthesis might be possible for narrower subsets. However, the largest categories, such as cheating and plagiarism, still combined different operationalisations and statistical formats, while smaller categories such as contract cheating, AI assisted misconduct, fabrication/falsification, and sadism specific findings contained too few comparable studies for meaningful pooling. Therefore, a structured narrative synthesis was the most methodologically appropriate option for the present evidence base. This follows established practice in adjacent literature examining dark traits and behavioural outcomes, where narrative synthesis is the dominant approach when multi model statistical heterogeneity is present [28, 96]. This heterogeneity does mean that cross study comparisons of trait strength should be interpreted cautiously, as studies did not always assess equivalent trait constructs, equivalent misconduct outcomes, or equivalent statistical parameters. As the evidence base grows and more studies use comparable measures and reporting formats, future reviews should consider conducting quantitative meta-analyses to estimate the overall strength and consistency of associations between dark personality traits and academic misconduct.

A further limitation concerns the conceptual and empirical overlap between Machiavellianism and psychopathy. Some research has questioned whether Machiavellianism is sufficiently distinct from psychopathy, arguing that the two constructs may capture overlapping antagonistic and manipulative tendencies [136]. This is relevant to the present synthesis because associations attributed to Machiavellianism may reflect shared variance with psychopathy or a broader dark core, particularly when brief Dark Triad measures are used [44, 136]. At the same time, other research argues that Machiavellianism may remain useful as a distinct construct because it is more strongly characterised by strategic calculation, impulse control, and context sensitive response to punishment risk, whereas psychopathy is more closely linked to impulsivity and reduced punishment sensitivity [137]. Accordingly, findings that distinguish Machiavellianism from psychopathy in the present review should be interpreted cautiously and treated as dependent on the measurement approach used in the primary studies.

We also acknowledge that the inclusion criterion requiring studies to assess at least the full Dark Triad, and where available the Dark Tetrad, narrowed the evidence base. This was a deliberate scope decision intended to preserve cross-trait comparability and construct alignment in co-measured designs. However, this means that single trait studies were excluded, including potentially relevant work on everyday sadism, primary and secondary psychopathy, and grandiose and vulnerable narcissism. This may have limited the breadth of evidence available for interpreting trait specific mechanisms and may partly explain the relatively small number of sadism findings. As a result, any conclusions about the added value of sadism within the Dark Tetrad framework should be treated as preliminary rather than definitive. Furthermore, the present synthesis should be interpreted as a review of dark personality clusters in academic misconduct contexts rather than an exhaustive synthesis of all individual dark trait literatures. Complementary reviews focused on individual traits or trait subtypes would be valuable for clarifying mechanisms that could not be fully addressed within the present comparative Triad/Tetrad framework.

Additionally, although used in a supplementary capacity, Google Scholars indexing boundaries and ranking algorithms limit reproducibility, which we acknowledge as a methodological constraint. Screening only the first 200 Google Scholar records may have missed relevant lower-ranked studies, particularly if ranking order changed over time or differed by search context. Web of Science could also not be searched due to institutional licensing restrictions. Future systematic reviews should include Web of Science when available. Although Scopus was used as a multidisciplinary supplementary database and exhibits extensive overlap with Web of Science in terms of citation coverage and indexing of psychology and education research [138], the absence of Web of Science means that some potentially relevant records may not have been captured. These search constraints should be considered when interpreting the comprehensiveness and reproducibility of the review.

Future research may also look at potential interventions or prevention strategies to reduce academic misconduct, with an avenue that may benefit from being informed by trait related risk patterns. For psychopathy, approaches that build empathy and impulse control could be useful, while for Machiavellianism, structural measures such as assessment redesign and plagiarism detection may limit opportunities for manipulation. Addressing academic entitlement in narcissism and fostering a culture of integrity to counter sadism may further reduce misconduct. If future research is able to replicate the present synthesis’ preliminary associations and extend them, specific interventions or strategies could be implemented to reduce academic misconduct risk among those with different trait profiles.

## Conclusions

This review provides a systematic narrative synthesis of evidence linking dark personality traits to academic misconduct. Across the included studies, all four traits were implicated in at least some academic misconduct related outcomes, although the strength and consistency of these associations varied and should be interpreted cautiously given heterogeneity in measures, outcomes and statistical models. Psychopathy showed the most consistent pattern of association across academic misconduct behaviours such as plagiarism, cheating, and contract cheating attitudes although future research may need to consider the difference in subtypes of psychopathy for a more defined explanation of behaviour. Narcissism, by contrast, showed weaker associations that were often dependent on context or situational factors.

Machiavellianism showed a nuanced profile, it was associated with behaviours such as plagiarism and exam cheating, but did not consistently relate to contract cheating, suggesting that not all forms of dishonesty align equally with its calculated, strategic characteristics. Sadism was investigated less frequently than the Dark Triad traits, while preliminary evidence linked sadism to lying in academic contexts and AI-assisted cheating, the small number of studies means that conclusions about its contribution to the Dark Tetrad framework remain tentative. Future research is needed until its full contribution can be evaluated.

Overall, the findings suggest that each Dark Tetrad trait may relate to academic misconduct behaviour in distinct ways when measured together, but these patterns should be interpreted as preliminary and conditional on the limitations of the evidence base. There is preliminary support that the Dark Tetrad provides a more comprehensive lens for understanding dishonest behaviours in academic settings than the Triad alone. Nonetheless, these conclusions should be drawn cautiously given differences in measurement approaches and the uneven coverage of specific misconduct behaviours across studies. This systematic review and narrative synthesis suggests the Dark Tetrad may offer a useful conceptual framework for integrity research om higher education, particularly if future studies assess all four traits using comparable measures and further distinguish between different forms of misconduct.

## Supplementary Information

Additional file 1.

Additional file 2.

## Data Availability

The datasets generated and/or analysed during the current study are available in the Open Science Framework repository, [https://osf.io/46ng2] (https://osf.io/46ng2) .

## Footnotes

1 The term Dark Tetrad or Dark Triad refers to the subclinical manifestation of narcissism, psychopathy, Machiavellianism, and sadism. For the remainder of the paper, when these terms are mentioned, it is referring to the non-clinical traits.

2 This article reported three studies, two met inclusion criteria and were extracted, one was excluded during full-text screening [106].

3 Note: The term “academic fraud” used in Srirejeki et al. (2023) refers to behaviours conceptually aligned with the umbrella term academic misconduct in the present review. Original terms presented in the articles are retained for transparency.

## Abbreviations

SD3: Short Dark Triad
SD4: Short Dark Tetrad
DTDD: Dirty Dozen
