# Supplementary material for: The Dark Tetrad and academic dishonesty: a systematic review and narrative synthesis of personality predictors of cheating, plagiarism, and deception in education
Source: BMC Psychol. 2026 May 29;14:1120. doi: 10.1186/s40359-026-04894-8 (PMC13422123; doi:10.1186/s40359-026-04894-8)
Supplement: Supplementary file 1 — Additional file 1. [file 40359_2026_4894_MOESM1_ESM.docx]

**Table 2.**

*Synthesis of Key Findings Derived from the Studies Examined in the Review*

| **Author (Year)** | **Country** | ***N*** | **Sample Characteristics**  **Age: *M* (*SD*)**  **Gender Distribution** | **Measure of Triad or Tetrad** | **Type of Academic Misconduct Measured** | **Main Findings** | **Limitations** | **Quality Rating (/20)** |
| --- | --- | --- | --- | --- | --- | --- | --- | --- |
| (Baughman et al., 2014) | Canada | 462 | 19.49 (4.86)  71.87% female | The Short Dark Triad (SD3; Jones & Paulhus, 2014) | Dishonesty | All traits positively correlated with how often lie in academic context (narcissism: *r*=0.14; Machiavellianism: *r*=0.25; psychopathy: *r*=0.19), positive emotions when lying (narcissism: *r*=0.28; Machiavellianism: *r*=0.33; psychopathy: *r*=0.42), how likely they think the lie to be believed (narcissism: *r*=0.19; Machiavellianism: *r*=0.16; psychopathy: *r*=0.17). Only Machiavellianism (*r*=0.28) and psychopathy (*r*=0.10) positively correlated with how much effort they would put into lie. When controlling for sex differences, only Machiavellianism positively predicted how often they lie (*β*=.18) and how much effort to lie (*β*=.29). Only narcissism predicted whether the lie would be believed (*β*=.14). All three predicted positive emotions when lying (narcissism: *β*=.14; Machiavellianism: *β*=.16; psychopathy: *β*=.19) | Self-report bias; university sample not representative | 17 |
| (Cheung & Egan, 2021) | United Kingdom | 252 | 21.88 (3.42)  54% female | SD3 | Cheating | All three traits positively correlated with scholastic cheating, (Machiavellianism: *r*=0.31; narcissism: *r*=0.21; psychopathy: *r*=0.63) | Self-report bias; scales used culturally bias; university sample not representative | 18 |
| (Clemente et al., 2025) | Spain | 912 | 36.89 (16.89)  62% female | The Short Dark Tetrad (SD4; Paulhus et al., 2018) | Cheating | All four traits showed a positive correlation with cheating (Machiavellianism: *r*=.25; narcissism: *r*=.17; psychopathy: *r*=.25; sadism: *r*=.09). Linear regressions showed Machiavellianism (*β*=.20) to be a positive predictor of cheating, whereas sadism (*β*=-.15) was a significant negative predictor. Narcissism and psychopathy were not significant. | Social desirability bias; recall bias | 15 |
| (Curtis, 2023) | Australia | 118 Australian students, +310 Prolific= 428 (390 after data cleaning) | NA*  67.69% female | SD3 | Plagiarism, Cheating | All three traits positively correlated with academic misconduct (psychopathy: *r*=0.35; narcissism: *r*=0.17; Machiavellianism: *r*=0.28) | Self-report bias; low internal consistency reliability of measures | 17 |
| (Curtis et al., 2022) | Australia | 387 | 25.71 (9.31)  80.10% female | SD3 | Plagiarism, Cheating | All three traits predicted academic misconduct (psychopathy: *r*=0.16; narcissism: *r*=0.14; Machiavellianism: *r*=0.14), mediation analysis showed no direct effect with any but were all mediated through academic entitlement scale of externalised responsibility | Self-report bias | 17 |
| (Esteves et al., 2021) | Brazil | 343 | 22.4 (5.52)  65.6% female | The Dirty Dozen (DTDD; Jonason & Webster, 2010) | Cheating | Dark Triad traits predicted the scores of engaging someone in cheating (*R²* =0.17, *F* (4.30) = 22.72, *p* < 0.001). Machiavellianism (*β*=.28) and narcissism (*β*=.16) positively predicted academic cheating, psychopathy not significant. The Dark Triad traits predicted the scores of taking the initiative in cheating (*R²* =0.15, *F* (14, 951) = 19.97, *p* < 0.001), Machiavellianism (*β*=.28) and narcissism (*β*=.17) were significant. | Self-report bias; academic performance not controlled for | 15 |
| (Forsyth et al., 2021) | Australia | 615 | 26.83 (8.36)  60.5% female | SD3; Short Sadistic Impulse Scale (O'Meara et al., 2011) | Dishonesty | All Dark Tetrad traits were positively related with a propensity to lie in an academic setting (Machiavellianism: *r*=.39; narcissism: *r*=.19; psychopathy: *r*=.23; sadism: *r*=.28), lying efficacy (Machiavellianism: *r*=.35; narcissism: *r*=.31; psychopathy: *r*=.24; sadism: *r*=.26), experience of positive emotion when lying (Machiavellianism: *r*=.39; narcissism: *r*=.34; psychopathy: *r*=.29; sadism: *r*=.39). All Dark Tetrad traits negatively correlated with the cognitive load (difficulty, effort to lie, and preparation) of lying (Machiavellianism: *r*=-.19; narcissism: *r*=-.18; psychopathy: *r*=-.27; sadism: *r*=-.29) and experiencing negative emotions when lying (Machiavellianism: *r*=-.20; narcissism: *r*=-.09; psychopathy: *r*=-.17; sadism: *r*=-.19). Only Machiavellianism (*β*=.31) and sadism (*β*=.12) positively predicted lying in an academic setting. | Self-report bias; uneven gender and age distribution; university sample not representative | 17 |
| (Greitemeyer & Kastenmüller, 2023) | Austria | 283 | 25 (7.75)  65.72% female | SD3 | Use of AI to cheat | All Dark Triad traits positively correlated with the intention to use ChatGPT to cheat (Machiavellianism: *r*=.13; narcissism: *r*=.20; psychopathy: *r*=.24) When accounting for the HEXACO traits, the dark traits also predicted chatbot intention use *F*(9, 282) = 4.64, *p* < .001, *R²* = 0.13. When controlling for the perceived quality, only narcissism (*β*=.18) and psychopathy (*β*=.21) were significant. Machiavellianism was not significant. | Homogeneity of sample culturally and university; self-report bias | 19 |
| (He et al., 2023) | China | 1201 | NA*  53.9% female | DTDD | Cheating | Machiavellianism (*r*=.32) and psychopathy (*r*=.26) were positively related to academic cheating, narcissism was not significant. Path analysis showed that performance avoidance partially mediated the effects of psychopathy and Machiavellianism on academic cheating (mediation = .06, *SE* = .02, 95% CI [.01, .08]/[.01, .09]), and a full mediator for narcissism (mediation = .05, *SE* = .02, 95% CI [.01, .08]); model fit was strong. | Self-report bias | 15 |
| (Kokkinos & Antoniadou, 2024) | Greece | 587 | 20.59 (3.11)  72.91% female | SD3 | Cheating, Plagiarism | Both Machiavellianism (*r*=.17) and psychopathy (*r*=.27) positively related to cheating, unauthorised collaboration (Machiavellianism: *r*=.17; psychopathy *r*=.17), and plagiarism (Machiavellianism: *r*=.13; psychopathy: *r*=.19). Narcissism was not significant. The path from psychopathy to unauthorised collaboration was moderated by moral disengagement (b = –0.12, *SE* = 0.04, 95% CI [–0.20, –0.03], *p* < .05). | University sample not representative; treats traits as unidimensional; lower internal consistency reliability scores with the SD3; future studies should consider influence of cheating peers and classroom environment | 15 |
| (Koscielniak et al., 2024) | Poland | 397 | 22.37 (3.40)  58.9% female | DTDD | Dishonesty | Machiavellianism (*r*=.27), narcissism (*r*=.22), and psychopathy (*r*=.18) positively correlated with individual exam dishonesty. Only Machiavellianism (*r*=.10) positively correlated with collective exam dishonesty. Machiavellianism (*r*=.21) and narcissism (*r*=.17), and psychopathy (*r*=.12) all positively correlated with a composite exam dishonesty score. | Self-report bias; homogeneity of sample culturally and university | 19 |
| (Lingán-Huamán et al., 2024) | Peru | 591 | 21.5 (3.60)  71.7% female | DTDD | Dishonesty | Several direct effects for dark triad traits and academic dishonesty were significant for male and female (Machiavellianism → cheating: male=.65 female=.69; Machiavellianism → plagiarism: male= .19 female=.21; psychopathy → plagiarism: male=.41 female= .26; Machiavellianism → falsification: male=.23 female=.60). Only psychopathy → falsification and significant for male (.39). There were no significant effects when mediated by moral disengagement. | Homogeneity of university sample; self-report bias; traits treated as unidimensional; cover more facets of academic dishonesty | 17 |
| (Mungall et al., 2025) | Canada | 535 | 20.16 (4)  81.5% female | Self-Report Psychopathy Scale 4-Short Form (Paulhus et al., 2009), Five Factor Machiavellianism Inventory (Collison et al., 2018), Narcissistic Grandiosity Scale (Rosenthal et al., 2020), Narcissistic Vulnerability Scale (Crowe et al., 2018) | Cheating | In a binary logistic regression model, the Machiavellianism item Agency was a negative predictor (LnOR -.41) and Planfulness (LnOR .39) a positive predictor of past cheating behaviour. The psychopathy item Interpersonal Manipulation (LnOR .45) was a positive predictor, vulnerable narcissism (LnOR .41) was also a positive predictor. In regressions, only grandiose narcissism (*β*=.11) was a positive predictor of endorsing cheating behaviour whereas the psychopathy item Affective *β*=-.27) was a negative predictor. | Homogeneity of sample, culturally and university; self-report bias | 20 |
| (Rundle et al., 2023) | Australia | 403 | 24.14 (8.36)  84.1% female | SD3 | Contract Cheating | Machiavellianism was positively related to not contract cheating because of fear of detection (*τ*=.22), self-efficacy and mistrust of others (*τ*=.18), a lack of opportunity (*τ*=.13), and barriers to consideration (*τ*=.22). Psychopathy was negatively related to morals and norms (*τ*=-.16) and moral alignment (*τ*=-.15). Narcissism was not significant. Psychopathy had a negative prediction of morals and norms (*β*=-.16). Machiavellianism was the strongest positive predictor for fear of detection and punishment (*β* =.42), self-efficacy and mistrust of others (*β* =.21), lack of opportunity (*β* =.10), and barriers to consideration (*β* =.25). | Uneven gender distribution | 15 |
| (Srirejeki et al., 2023) | Indonesia | 259 | NA*  71.04% female | SD3 | Academic Fraud | Partial Least Squares analysis showed the dark triad (*β* =.35) to be a significant predictor of academic fraud intention. Machiavellianism (*β* =.12), narcissism (*β* =.10), and psychopathy (*β* =.20) all had a positive and significant effect on student’s intention to engage in academic fraud. | University sample not representative; situational variables not moderated | 15 |
| (Stojanov et al., 2025) | United States | 319 | 25.83 (8.13)  48.34% female | SD3 | Misconduct | All three dark triad traits were positively related to academic misconduct (Machiavellianism: *r*=.37; narcissism: *r*=.22; psychopathy: *r*=.32). PROCESS analysis revealed non-significant interactions between the dark triad traits, SCACCIA Communication, and academic misconduct. A follow up simple slope analysis revealed higher levels of SCACCIA Communication weakened the effect of psychopathy (+1 SD of SACCIA communication *β* = 0.20, *p* < .01; *SE* not reported) and marginally for narcissism (+1 SD of SACCIA communication *β* = 0.11, *SE* = 0.06, *p* = .07) on academic misconduct. | Self-report bias; does not address cultural or motivational aspects of academic misconduct; SACCIA framework lacks refinement; university sample not representative | 15 |
| (Sun et al., 2025) | Taiwan | 812 | 24.86 (5.98)  54.06% female | SD4 | Use of AI to cheat | Narcissism (*r*=.33), psychopathy (*r*=.37), and sadism (*r*=.34) all positively related to generative AI academic misconduct. Machiavellianism was not significant. In hierarchical multiple regressions, these three traits all positively predicted generative AI academic misconduct (narcissism: *β* =.20; psychopathy: *β* =.19; sadism: *β* =.18). Machiavellianism was not significant. | Pilot validation of the GAIAM scale was not undertaken to ensure scale reliability. Self-report bias. Culturally homogenous sample. | 15 |
| (Ternes et al., 2019) | Canada | 330 | 21.5 (4.1)  78.8% female | SD3; The Levenson’s Self-Report Psychopathy Scale (Levenson et al., 1995) | Plagiarism, Cheating, Misconduct | Psychopathy was positively related to several academic misconduct variables (high risk: *r*=.19; fabrication: *r*=.12; low risk: *r*=.16; academic misconduct total: *r*=.18) as was Machiavellianism (high risk: *r*=.12; fabrication: *r*=.13; low risk: *r*=.12; academic misconduct total: *r*=.12). Primary psychopathy was positively related to every academic misconduct variable (high risk: *r*=.27; copying: *r*=.15; plagiarism: *r*=.22; fabrication: *r*=.30; low risk: *r*=.20; academic misconduct total: *r*=.30) with secondary psychopathy also significant with several variables (high risk: *r*=.17; low risk: *r*=.14; academic misconduct total: *r*=.16). Narcissism was not significantly related to any variables. After controlling for impulsivity, psychopathy was a positive predictor of high risk (*β* =.15) and academic misconduct total (*β* =.14). Primary psychopathy was a positive predictor of several variables (high risk: *β* =.23; copying: *β* =.13; plagiarism: *β* =.22; falsification: *β* =.33; low risk: *β* =.14; academic misconduct total: *β* =.28) Machiavellianism and secondary psychopathy were not significant. | Self-report bias. Gender imbalance, small effects sizes that further variables may explain a large variance of the data. | 16 |
| (Turnipseed & Landay, 2018) | United States | 519 | 21.4 (2.5)  51% female | DTDD | Cheating | Machiavellianism (*r*=.20) and Psychopathy (*r*=.12) were positively related to academic cheating. Machiavellianism (*β* =.03) remained the only positive predictor of academic cheating when controlling for age, race, and gender. | Self-Report bias. Psychopathy treated as a unidimensional construct may explain non-significance. | 16 |
| (Veríssimo et al., 2022) | Portugal | 591 | 20.4 (3.2)  66.7% female | DTDD | Misconduct | All three dark triad traits were positively related to academic misconduct, (Machiavellianism: *r*=.29; narcissism: *r*=.11; psychopathy: *r*=.09; dirty dozen total: *r*=.23). Machiavellianism (*β* =.28) remained the only dark trait that positively predicted academic misconduct when controlling for academic year, peer fraud, severity of penalty, psychological well-being, age, and gender. | Self-report bias, homogenous sampling. | 19 |
| (Williams et al., 2010) Study 1 | United States | 249 | NA*  70% female | Narcissistic Personality Inventory (Raskin & Terry, 1988); MACH IV (Christie & Geis, 2013); Self-Report Psychopathy Scale-III (Williams et al., 2003) | Cheating | All three dark triad traits correlated with academic cheating (Machiavellianism: *r*=.39; narcissism: *r*=.20; psychopathy: *r*=.58). After controlling for other predictors, only psychopathy (*β* = .50) remained a significant positive predictor. | Self-report bias. | 15 |
| (Williams et al., 2010) Study 2 | United States | 107 | NA*  67.3% female | NPI; MACH IV; SRP-III | Plagiarism | All three dark triad traits correlated with TurnitIn plagiarism scores (Machiavellianism: *r*=.14; narcissism: *r*=.12; psychopathy: *r*=.22). | Self-report bias, small sample size. Low frequency of identified plagiarists. | 15 |
| (Zhang et al., 2019) | China | 634 | 19.95 (1.04)  83.43% female | SD3 | Cheating | All three dark triad traits were positively correlated with academic cheating behaviour (Machiavellianism: *r*=.12; narcissism: *r*=.15; psychopathy: *r*=.21). Only psychopathy (*β* =.40) remained a significant positive predictor when controlling for the Big Five, gender, and age. | Gender imbalance and culturally homogenous. Other factors such as honesty-humility and cognitive ability that negatively predict cheating could explain further variance. | 15 |

*Note.* * Data not provided in article

Baughman, H. M., Jonason, P. K., Lyons, M., & Vernon, P. A. (2014). Liar liar pants on fire: Cheater strategies linked to the Dark Triad. *Personality and Individual Differences*, *71*, 35-38. <https://doi.org/https://doi.org/10.1016/j.paid.2014.07.019>

Cheung, Y. K., & Egan, V. (2021). The HEXACO-60, the Dark Triad and scholastic cheating. *Psychological Reports*, *124*(6), 2774-2794. <https://doi.org/https://doi.org/10.1177/0033294120961071>

Christie, R., & Geis, F. L. (2013). *Studies in machiavellianism*. Academic Press. <https://books.google.com.au/books?hl=en&lr=&id=d5tGBQAAQBAJ&oi=fnd&pg=PP1&dq=Studies+in+machiavellianism++R.+Christie+and+F.+L.+Geis++++Publisher:+Academic+Press+2013+&ots=rknIGLOHA0&sig=97P8uXBy-DLXFkJ8D7uarhK0VjU&redir_esc=y#v=onepage&q&f=false>

Clemente, M., Espinosa, P., Aguilar-Valera, J. A., & Casado-Patricio, E. (2025). Cheating by university students and its relationship to Dark Personality. *Revista de Estudios e Investigación en Psicología y Educación*, *12*(2), e12608-e12608.

Collison, K. L., Vize, C. E., Miller, J. D., & Lynam, D. R. (2018). Development and preliminary validation of a five factor model measure of Machiavellianism. *Psychological assessment*, *30*(10), 1401.

Crowe, M. L., Edershile, E. A., Wright, A. G., Campbell, W. K., Lynam, D. R., & Miller, J. D. (2018). Development and validation of the Narcissistic Vulnerability Scale: An adjective rating scale. *Psychological assessment*, *30*(7), 978.

Curtis, G. J. (2023). It Kant be all bad: contributions of light and dark triad traits to academic misconduct. *Personality and Individual Differences*, *212*, 112262. <https://doi.org/https://doi.org/10.1016/j.paid.2023.112262>

Curtis, G. J., Correia, H. M., & Davis, M. C. (2022). Entitlement mediates the relationship between dark triad traits and academic misconduct. *Personality and Individual Differences*, *191*, 111563. <https://doi.org/https://doi.org/10.1016/j.paid.2022.111563>

Esteves, G. G. L., Oliveira, L. S., de Andrade, J. M., & Menezes, M. P. (2021). Dark triad predicts academic cheating. *Personality and Individual Differences*, *171*, 110513. <https://doi.org/https://doi.org/10.1016/j.paid.2020.110513>

Forsyth, L., Anglim, J., March, E., & Bilobrk, B. (2021). Dark Tetrad personality traits and the propensity to lie across multiple contexts. *Personality and Individual Differences*, *177*, 110792. <https://doi.org/https://doi.org/10.1016/j.paid.2021.110792>

Greitemeyer, T., & Kastenmüller, A. (2023). HEXACO, the Dark Triad, and Chat GPT: Who is willing to commit academic cheating? *Heliyon*, *9*(9). <https://doi.org/https://doi.org/10.1016/j.heliyon.2023.e19909>

He, Q., Zheng, Y., Yu, Y., & Zhang, J. (2023). The dark triad, performance avoidance, and academic cheating. *PsyCh Journal*, *12*(3), 461-463. <https://doi.org/https://doi.org/10.1002/pchj.632>

Jonason, P. K., & Webster, G. D. (2010). The dirty dozen: a concise measure of the dark triad. *Psychological assessment*, *22*(2), 420. <https://doi.org/https://psycnet.apa.org/doi/10.1037/a0019265>

Jones, D. N., & Paulhus, D. L. (2014). Introducing the short dark triad (SD3) a brief measure of dark personality traits. *Assessment*, *21*(1), 28-41. <https://doi.org/https://doi.org/10.1177/1073191113514105>

Kokkinos, C. M., & Antoniadou, N. (2024). Understanding academic dishonesty in university settings: The interplay of dark triad traits and moral disengagement. *The Journal of Genetic Psychology*, *185*(5), 309-322. <https://doi.org/https://doi.org/10.1080/00221325.2023.2297850>

Koscielniak, M., Enko, J., & Gąsiorowska, A. (2024). “I Cheat” or “We Cheat?” The structure and psychological correlates of individual vs. Collective examination dishonesty. *Journal of Academic Ethics*, *22*(1), 71-87.

Levenson, M. R., Kiehl, K. A., & Fitzpatrick, C. M. (1995). Assessing psychopathic attributes in a noninstitutionalized population. *Journal of Personality and Social Psychology*, *68*(1), 151. <https://psycnet.apa.org/buy/1995-17458-001>

Lingán-Huamán, S. K., Dominguez-Lara, S., & Esteban, R. F. C. (2024). Gender-based differences in the impact of Dark Triad traits on academic dishonesty: The mediating role of moral disengagement in college students. *Heliyon*, *10*(1). <https://doi.org/https://doi.org/10.1016/j.heliyon.2023.e23322>

Mungall, L. R., Fazaa, G. R., & Blais, J. (2025). A Pre-Registered Examination of the Relationship Between Personality, Stress, and Academic Cheating in the Age of Online Learning. *Psychological Reports*, 00332941251379435.

O'Meara, A., Davies, J., & Hammond, S. (2011). The psychometric properties and utility of the Short Sadistic Impulse Scale (SSIS). *Psychological assessment*, *23*(2), 523. <https://doi.org/https://psycnet.apa.org/doi/10.1037/a0022400>

Paulhus, D., Buckels, E., Trapnell, P., & Jones, D. (2018). Then there were four: The Short Dark Tetrad (SD4). *Manuscript under review]. University of British Columbia*.

Paulhus, D. L., Neumann, C. S., & Hare, R. D. (2009). Manual for the self-report psychopathy scale. In: Toronto: Multi-health systems.

Raskin, R., & Terry, H. (1988). A principal-components analysis of the Narcissistic Personality Inventory and further evidence of its construct validity. *Journal of Personality and Social Psychology*, *54*(5), 890. <https://psycnet.apa.org/buy/1988-25254-001>

Rosenthal, S. A., Hooley, J. M., Montoya, R. M., van der Linden, S. L., & Steshenko, Y. (2020). The Narcissistic Grandiosity Scale: A measure to distinguish narcissistic grandiosity from high self-esteem. *Assessment*, *27*(3), 487-507.

Rundle, K., Curtis, G. J., & Clare, J. (2023). Why students do not engage in contract cheating: A closer look. *International Journal for Educational Integrity*, *19*(1), 11. <https://doi.org/https://doi.org/10.3389/fpsyg.2019.02229>

Srirejeki, K., Faturokhman, A., Praptapa, A., & Irianto, B. S. (2023). Understanding academic fraud: the role of dark triad personality and situational factor. *Journal of Criminal Justice Education*, *34*(2), 147-168. <https://doi.org/https://doi.org/10.1080/10511253.2022.2068630>

Stojanov, A., Hannawa, A., & Adam, L. (2025). Communication Following the SACCIA Framework may Weaken the Relationship Between the Dark Triad and Academic Misconduct. *Communication reports*, *38*(1), 1-12. <https://doi.org/https://doi.org/10.1080/08934215.2024.2424535>

Sun, R., Tang, M., Loan, N. T. T., Zhou, J., & Wang, C.-Y. (2025). The dark tetrad as associated factors in generative AI academic misconduct: insights beyond personal attribute variables. Frontiers in Education,

Ternes, M., Babin, C., Woodworth, A., & Stephens, S. (2019). Academic misconduct: An examination of its association with the dark triad and antisocial behavior. *Personality and Individual Differences*, *138*, 75-78. <https://doi.org/https://doi.org/10.1016/j.paid.2018.09.031>

Turnipseed, D. L., & Landay, K. (2018). The role of the dark triad in perceptions of academic incivility. *Personality and Individual Differences*, *135*, 286-291. <https://doi.org/https://doi.org/10.1016/j.paid.2018.07.029>

Veríssimo, A. C., Conrado, G. A., Barbosa, J., Gomes, S. F., Severo, M., Oliveira, P., & Ribeiro, L. (2022). Machiavellian medical students report more academic misconduct: A cocktail fuelled by psychological and contextual factors. *Psychology Research and Behavior Management*, 2097-2105. <https://doi.org/https://doi.org/10.2147/PRBM.S370402>

Williams, K. M., Nathanson, C., & Paulhus, D. L. (2003). Structure and validity of the self-report psychopathy scale-III in normal populations. 111th annual convention of the American Psychological Association,

Williams, K. M., Nathanson, C., & Paulhus, D. L. (2010). Identifying and profiling scholastic cheaters: their personality, cognitive ability, and motivation. *Journal of experimental psychology: applied*, *16*(3), 293. <https://doi.org/https://psycnet.apa.org/doi/10.1037/a0020773>

Zhang, J., Paulhus, D. L., & Ziegler, M. (2019). Personality predictors of scholastic cheating in a Chinese sample. *Educational Psychology*, *39*(5), 572-590. <https://doi.org/https://doi.org/10.1080/01443410.2018.1502414>
