# Supplementary material for: The Dark Tetrad and academic dishonesty: a systematic review and narrative synthesis of personality predictors of cheating, plagiarism, and deception in education
Source: BMC Psychol. 2026 May 29;14:1120. doi: 10.1186/s40359-026-04894-8 (PMC13422123; doi:10.1186/s40359-026-04894-8)
Supplement: Supplementary file 2 — Additional file 2. [file 40359_2026_4894_MOESM2_ESM.docx]

Table S1

*Quality Assessment of the Studies Included in the Current Systematic Literature Review (Questions 1 – 10)*

| Author (Year) | Q1. | Q2. | Q3. | Q4. | Q5. | Q6. | Q7. | Q8. | Q9. | Q10. | Total Quality Rating /20 |
| --- | --- | --- | --- | --- | --- | --- | --- | --- | --- | --- | --- |
| Baughman et al. (2014) | 1 | 1 | 0 | 1 | 1 | 1 | 0 | 1 | 1 | 1 | 17 |
| Cheung and Egan (2021) | 1 | 1 | 1 | 1 | 1 | 1 | 0 | 1 | 1 | 1 | 18 |
| Clemente et al. (2025) | 1 | 1 | 0 | 1 | 1 | 1 | 0 | 1 | 1 | 1 | 15 |
| Curtis (2023) | 1 | 1 | 0 | 1 | 1 | 1 | 1 | 1 | 1 | 1 | 17 |
| Curtis, Correia, and Davis (2022) | 1 | 1 | 0 | 1 | 1 | 1 | 1 | 1 | 1 | 1 | 17 |
| Esteves et al. (2021) | 1 | 1 | 0 | 1 | 1 | 0 | 0 | 1 | 1 | 1 | 15 |
| Forsyth et al. (2021) | 1 | 1 | 1 | 1 | 0 | 0 | 0 | 1 | 1 | 1 | 17 |
| Greitemeyer and Kastenmüller (2023) | 1 | 1 | 1 | 1 | 1 | 1 | 0 | 1 | 1 | 1 | 19 |
| He et al. (2023) | 1 | 1 | 0 | 1 | 1 | 0 | 0 | 1 | 1 | 1 | 15 |
| Kokkinos and Antoniadou (2024) | 1 | 1 | 0 | 1 | 0 | 0 | 0 | 1 | 1 | 1 | 15 |
| Koscielniak, Enko, and Gąsiorowska (2024) | 1 | 1 | 0 | 1 | 1 | 1 | 1 | 1 | 1 | 1 | 19 |
| Lingán-Huamán, Dominguez-Lara, and Esteban 2024) | 1 | 1 | 1 | 1 | 1 | 0 | 0 | 1 | 1 | 1 | 17 |
| Mungall, Fazaa, and Blais (2025) | 1 | 1 | 1 | 1 | 1 | 1 | 1 | 1 | 1 | 1 | 20 |
| Rundle, Curtis, and Clare (2019) | 1 | 1 | 0 | 1 | 0 | 0 | 0 | 1 | 1 | 1 | 15 |
| Srirejeki et al. (2023) | 1 | 1 | 1 | 1 | 0 | 0 | 0 | 1 | 1 | 1 | 15 |
| Stojanov, Hannawa, and Adam (2025) | 1 | 1 | 1 | 1 | 0 | 0 | 0 | 1 | 1 | 1 | 15 |
| Sun et al. (2025) | 1 | 1 | 0 | 1 | 0 | 0 | 0 | 1 | 1 | 1 | 15 |
| Ternes et al. (2019) | 1 | 1 | 0 | 1 | 1 | 0 | 0 | 1 | 1 | 1 | 16 |
| Turnipseed and Landay (2018) | 1 | 1 | 0 | 1 | 1 | 1 | 0 | 1 | 1 | 1 | 16 |
| Veríssimo et al. (2022) | 1 | 1 | 0 | 1 | 1 | 1 | 1 | 1 | 1 | 1 | 19 |
| Williams, Nathanson, and Paulhus (2010) Study 1 | 1 | 1 | 0 | 1 | 1 | 1 | 0 | 1 | 1 | 1 | 15 |
| Williams, Nathanson, and Paulhus (2010) Study 2 | 1 | 1 | 0 | 1 | 1 | 1 | 0 | 1 | 1 | 1 | 15 |
| Zhang, Paulhus, and Ziegler (2019) | 1 | 1 | 0 | 1 | 1 | 0 | 0 | 1 | 1 | 1 | 15 |

Table S2

*Quality Assessment of the Studies Included in the Current Systematic Literature Review (Questions 11 – 20)*

| Author (Year) | Q11. | Q12. | Q13. ^[[1]](#footnote-1)^ | Q14. ^[[2]](#footnote-2)^ | Q15. | Q16. | Q17. | Q18. | Q19. ^[[3]](#footnote-3)^ | Q20. | Total Quality Rating /20 |
| --- | --- | --- | --- | --- | --- | --- | --- | --- | --- | --- | --- |
| Baughman et al. (2014) | 1 | 1 | 1 | 1 | 1 | 1 | 1 | 1 | 1 | 0 | 17 |
| Cheung and Egan (2021) | 1 | 1 | 1 | 1 | 1 | 1 | 1 | 1 | 1 | 0 | 18 |
| Clemente et al. (2025) | 1 | 1 | 0 | 1 | 1 | 1 | 0 | 1 | 1 | 1 | 15 |
| Curtis (2023) | 1 | 1 | 1 | 1 | 1 | 1 | 1 | 1 | 0 | 0 | 17 |
| Curtis, Correia, and Davis (2022) | 1 | 1 | 1 | 1 | 1 | 1 | 1 | 1 | 0 | 0 | 17 |
| Esteves et al. (2021) | 1 | 0 | 1 | 1 | 1 | 1 | 1 | 1 | 1 | 0 | 15 |
| Forsyth et al. (2021) | 1 | 1 | 1 | 1 | 1 | 1 | 1 | 1 | 1 | 1 | 17 |
| Greitemeyer and Kastenmüller (2023) | 1 | 1 | 1 | 1 | 1 | 1 | 1 | 1 | 1 | 1 | 19 |
| He et al. (2023) | 0 | 0 | 1 | 1 | 1 | 1 | 1 | 1 | 1 | 1 | 15 |
| Kokkinos and Antoniadou (2024) | 1 | 1 | 1 | 0 | 1 | 1 | 1 | 1 | 1 | 1 | 15 |
| Koscielniak, Enko, and Gąsiorowska (2024) | 1 | 1 | 0 | 1 | 1 | 1 | 1 | 1 | 1 | 1 | 19 |
| Lingán-Huamán, Dominguez-Lara, and Esteban 2024) | 1 | 1 | 1 | 0 | 1 | 1 | 1 | 1 | 1 | 1 | 17 |
| Mungall, Fazaa, and Blais (2025) | 1 | 1 | 1 | 1 | 1 | 1 | 1 | 1 | 1 | 1 | 20 |
| Rundle, Curtis, and Clare (2019) | 1 | 1 | 1 | 0 | 1 | 1 | 1 | 1 | 1 | 1 | 15 |
| Srirejeki et al. (2023) | 1 | 1 | 1 | 0 | 1 | 1 | 1 | 1 | 1 | 0 | 15 |
| Stojanov, Hannawa, and Adam (2025) | 1 | 1 | 1 | 0 | 1 | 1 | 1 | 1 | 1 | 1 | 16 |
| Sun et al. (2025) | 1 | 1 | 1 | 0 | 1 | 1 | 1 | 1 | 1 | 1 | 15 |
| Ternes et al. (2019) | 1 | 1 | 1 | 0 | 1 | 1 | 1 | 1 | 1 | 1 | 16 |
| Turnipseed and Landay (2018) | 1 | 1 | 1 | 1 | 1 | 1 | 1 | 1 | 0 | 0 | 16 |
| Veríssimo et al. (2022) | 1 | 1 | 1 | 1 | 1 | 1 | 1 | 1 | 1 | 1 | 19 |
| Williams, Nathanson, and Paulhus (2010) Study 1 | 1 | 1 | 1 | 0 | 1 | 1 | 1 | 1 | 0 | 0 | 15 |
| Williams, Nathanson, and Paulhus (2010) Study 2 | 1 | 1 | 1 | 0 | 1 | 1 | 1 | 1 | 0 | 0 | 15 |
| Zhang, Paulhus, and Ziegler (2019) | 1 | 1 | 1 | 0 | 1 | 1 | 1 | 1 | 1 | 0 | 15 |

Moor, L., & Anderson, J. R. (2019). A systematic literature review of the relationship between dark personality traits and antisocial online behaviours. *Personality and Individual Differences*, *144*, 40-55. <https://doi.org/https://doi.org/10.1016/j.paid.2019.02.027>

1. Q13 was reversed scored (i.e. 1 = *no* rather than 1 = *yes*) so that, if the study’s response rate did not indicate a risk of non-response bias, this would add positively to the overall quality rating of the study (Moor & Anderson, 2019). [↑](#footnote-ref-1)
2. Q14 was also reversed scored (i.e. 1 = *no* rather than 1 = *yes*) due to the same reason as detailed above. [↑](#footnote-ref-2)
3. Q19 was also reversed scored (i.e. 1 = *no* rather than 1 = *yes*) due to the same reason as detailed above. [↑](#footnote-ref-3)
